# Effects of antepartum hemorrhage on maternal and perinatal adverse outcomes in Northern Ethiopia: a retrospective cohort study

**DOI:** 10.1186/s12884-025-07829-0

**Published:** 2025-07-22

**Authors:** Mamit Gebreslassie Gebrekidan, Meseret Abay Fisseha, Aregawi Gebreyesus Belay, Girmatsion Fisseha Abreha

**Affiliations:** 1 Department of Reproductive Health, School of Public Health, College of Health Science, Mekelle University, Mekelle, Tigray, Ethiopia; 2 Department of Epidemiology, School of Public Health, College of Health Science, Mekelle University, Mekelle, Tigray, Ethiopia

**Keywords:** Antepartum hemorrhage, Maternal, Perinatal adverse outcomes, Mekelle, Ethiopia

## Abstract

**Background:**

Antepartum hemorrhage (APH) complicates 2–5% of all pregnancies and is the main cause of fetal and maternal death. However, little is known about the adverse maternal and perinatal outcomes of antepartum hemorrhage in the Tigray region. Therefore, this study assessed the effects of antepartum hemorrhage on maternal and perinatal adverse outcomes at Ayder Comprehensive Specialized Hospital, Mekelle, Tigray, Ethiopia, in 2024.

**Methods:**

An institutional-based retrospective cohort study was conducted among 539 mothers who gave birth between September 2019 and August 2021 at Ayder Comprehensive Specialized Hospital, Tigray, Ethiopia. Mothers who gave birth with and without antepartum hemorrhage were categorized as exposed and nonexposed groups, respectively. A systematic sampling method was used to select participants from medical records. Data were collected through a retrospective review of medical records. A modified Poison regression model with robust standard errors was used to estimate relative risk (RR). An adjusted relative risk (ARR) with 95% confidence interval (CI) and a *p*-value < 0.05 were considered statistically significant.

**Results:**

The incidence of adverse maternal outcomes among mothers with antepartum hemorrhage was 46.1%, compared to 14.2% among mothers without APH. Approximately 57.2% of mothers with APH and 18.9% of those without APH experienced adverse perinatal outcomes. Mothers with antepartum hemorrhage were more likely to experience postpartum hemorrhage (ARR = 3.9, 95% CI: 1.8, 8.8), emergency cesarean section (ARR = 2.9, 95% CI: 2.1, 3.9), preterm birth (ARR = 3.9, 95%CI: 2.8, 5.6), low birth weight baby (ARR = 4.5, 95%CI: 3.0, 6.6), stillbirth (ARR = 3.8, 95%CI: 1.9, 7.4), perinatal death (ARR = 3.7, 95%CI: 2.0, 6.9), admission to the NICU (ARR = 6.7, 95% CI: 3.1, 14.9), low Apgar score at the first minute (ARR = 2.8, 95%CI: 1.8, 4.3), and low Apgar score at the fifth minute (ARR = 3.7, 95% CI: 2.0, 6.8) compared to mothers without APH.

**Conclusion:**

Antepartum hemorrhage is associated with an increased risk of adverse maternal and perinatal outcomes; -including postpartum hemorrhage, emergency cesarean section, preterm birth, low birth weight, stillbirth, perinatal death, a low Apgar score and admission to the NICU. Improving access to emergency obstetric care in areas with a high burden of APH should be critical to ensure timely intervention and reduce adverse maternal and perinatal outcomes.

**Supplementary Information:**

The online version contains supplementary material available at 10.1186/s12884-025-07829-0.

## Background

Obstetric hemorrhage refers to excess bleeding at any time during pregnancy and delivery (antepartum hemorrhage) or after childbirth in the postpartum period (postpartum hemorrhage) [1]. Obstetric hemorrhage remain the major cause of maternal death and one of the leading obstetric causes of perinatal mortality across all age group, disproportionally, affecting women living in low-income countries [2].

Antepartum hemorrhage (APH) is defined as bleeding from the genital tract of the pregnant mother after the fetus has reached the age of viability (after 28 completed weeks and before delivery of the fetus) [3]. The causes of antepartum hemorrhage include placenta previa, abruption placenta and extraplacental. Placenta previa and abruption placenta account for more than half of all causes of antepartum hemorrhage. Placenta previa refers to when the placenta is implanted wholly or partially in the lower uterine segment [4]. Placenta abruption is the separation of the placenta either partially or completely from its implantation site before delivery [5]. The extraplacental causes may include cervical polyps, cervical erosions, endocervical erosions, cancer of the cervix, foreign bodies, genital lacerations and vasa previa [4].

Antepartum hemorrhage is an obstetric emergency associated with high maternal and perinatal morbidity and mortality. APH complicates 2–5% of pregnancies and is the major cause of fetal and maternal death [6].

In 2020, an estimated 287, 000 maternal deaths occurred globally, with nearly 95% reported in low-and middle-income countries. The African region alone accounted for 69% of these deaths, with a maternal mortality ratio of 531 deaths per 100,000 live births. Obstetric hemorrhage remains the leading direct cause of maternal mortality in these regions [7].

Studies conducted in both developed and developing countries have consistently shown that antepartum hemorrhage (APH) is associated with a range of adverse maternal and perinatal outcomes. Maternal complications include increased risk of postpartum hemorrhage (PPH), shock, retained placenta, cesarean section, coagulation failure, puerperal infections, and maternal death [8, 9]. Perinatal complications frequently reported in association with APH include premature delivery, low birth weight, intrauterine death, congenital malformations and birth asphyxia [6, 9]. A significant proportion of women with APH deliver low birth weight babies, often as a result of preterm labor or repeated episodes of bleeding that lead to prolonged placental inadequacy and fetal growth restriction [3].

Despite significant advancements in obstetric care, antepartum hemorrhage remains a persistent clinical challenge globally. It continues to be a major cause of maternal and perinatal morbidity and mortality, particularly in low- and middle-income countries, where delays in diagnosis, referral, and treatment are common due to limited resources and weak health systems [10].

In Ethiopia, despite inconsistent findings regarding the incidence of adverse maternal and perinatal outcomes associated with antepartum hemorrhage, the few available studies revealed a relatively high incidence of PPH, aneamia, perinatal mortality, preterm delivery, and low birth weight [3, 9]. However, little is known about the adverse maternal and perinatal outcomes of antepartum hemorrhage in the Tigray region. Moreover, the available studies conducted in Ethiopia did not fully address the associations between antepartum hemorrhage and maternal and perinatal adverse outcomes. The findings of this study can serve as an entry point to identify adverse outcomes of APH in mothers and neonates, and helping to plan the appropriate management strategies to improve maternal and neonatal health. Additionally, the result may inform policymakers and programmers designers in planning interventions aimed at improving care during pregnancy, delivery and after delivery ultimately reducing the adverse outcomes of APH. Therefore, the objective of this study was to assess the effect of antepartum hemorrhage on maternal and perinatal adverse outcomes at Ayder Comprehensive Specialized Hospital (ACSH).

## Methods

### Study area and period

Mekelle has one comprehensive specialized hospital, two general hospitals, two primary hospitals and eleven health centers and more than 15 private health institutions [11]. Mekelle is the capital city of Tigray. This study was conducted at Ayder Comprehensive Specialized Hospital in the Tigray region of Ethiopia. ACSH provides services such as surgical, gynecological, obstetric, medical, pediatrics, minor and major operation, and ophthalmologic and diagnostic facilities. ACSH is a public hospital with a capacity of 650 inpatient beds, 9 operating theatres, and more than 1500 health professionals in different fields of study and is also used as a teaching hospital, and research center for the College of Health Sciences at Mekelle University. As a tertiary hospital, it has a catchment population of up to five million people, serving the Tigray regions, the northern part of Afar region, and northeast of the Amhara region. The hospital has 13 departments. The labor ward provides delivery services on average for approximately 500 mothers per month [12, 13]. The study was conducted from October 01–October 30, 2021.

### Study design and population

An institutional-based retrospective cohort study was conducted through a document review of mothers with and without antepartum hemorrhage who gave birth at the ACSH between September 1, 2019, and August 2021. Two groups were included: mothers with antepartum hemorrhage (exposed group) and mothers without antepartum hemorrhage (nonexposed group) during the most recent pregnancy. Mother with multiple pregnancies, chronic medical illnesses and uterine rupture were excluded to avoid confounding effects. Mothers with incomplete medical records were also excluded from the study.

### Sample size determination and procedures

A double population proportion formula was used to calculate the sample size. The maximum sample for this study was calculated from the outcome variable low birth weight by considering the following assumptions: a two-sided confidence level of 95%, a power level of 80%, a ratio of exposed to nonexposed 1:2, P1 = the proportion of low birth weight among women with APH of 35.4% [14], and P2 = the proportion of low birth weight among women without APH of 23.4% [14]. After considering a 10% nonresponse rate, the final total sample size was 539 mothers. Among these, 180 mothers with antepartum hemorrhage during the most recent pregnancy and 359 mothers without antepartum hemorrhage during the most recent pregnancy were included in this study.

### Sampling procedures

Medical records were reviewed from the delivery registration log book of the labor ward. A systematic sampling method was employed to select mothers’ medical charts. Two different k^th^ values (sampling intervals) were calculated separately for the exposed and nonexposed groups. The total number of deliveries during the study period was 347 antepartum hemorrhages and 9272 without antepartum hemorrhages. We have a separate list for exposed (mothers with APH) and nonexposed (mothers without APH). K1 in the exposed group = total number of deliveries with APH during the study period/calculated sample size for antepartum hemorrhage, (347/180) = 1.93 approximately 2. Thus, every 2nd interval medical chart was selected from the registration log book. For the nonexposed group: K2 = total number of deliveries without antepartum hemorrhage during the study period/the calculated sample size without antepartum hemorrhage, (9272/359) = 26. Thus, every 26th interval medical charts were selected from the registration log book. The first chart was selected randomly for both groups from 1 to k^th^ intervals.

### Variables and measurement

The dependent variables of the study were maternal adverse outcomes (postpartum hemorrhage, admission to the ICU, maternal death, need for blood transfusion and emergency cesarean section) and perinatal adverse outcomes (preterm birth, LBW, stillbirth, perinatal deaths, low Apgar score, and admission to the NICU).

The independent variables included sociodemographic characteristics (age, residence), reproductive and obstetric factors (history of abortion, history of stillbirth, history of APH, history of cesarean section, gravidity, parity, antenatal care visit, gestational age at delivery, mode of delivery, and APH in the most recent pregnancy).

#### Adverse maternal outcomes

Were defined as any of the following; postpartum hemorrhage, admission to the ICU, maternal death [3], need for blood transfusion and emergency cesarean Section. [15].

#### Adverse perinatal outcomes

Were defined as the occurrence of any of the following: preterm birth, LBW, stillbirth, perinatal death, low Apgar score, and admission to the NICU [3]. The perinatal adverse outcome represents only the fetal/neonatal outcome.

#### Postpartum hemorrhage

Defined as blood loss of more than 500 milliliters (ml) following a vaginal delivery or more than 1000 ml following cesarean section delivery [16].

#### Preterm birth

Defined as a birth occurring at less than 37 completed weeks of gestation [17]. 

#### Perinatal death

Defined as fetal death that occurred in utero after a gestational age of 28 weeks or more and infant death that occurred at less than 7 days of age postdelivery [17].

#### Low birth weight

Defined as a birth weight of less than 2500 grams [18].

#### Low Apgar score

Defined as a newborn with an Apgar score of less than 7 at 1 and 5 minutes [18]. 

### Data collection tools and quality management

Data were collected from medical records using a checklist prepared by reviewing different studies [14, 17, 19, 20]. The checklist has four sections: a sociodemographic information, a reproductive and obstetric factor, an adverse maternal outcomes, and an adverse perinatal outcomes. Data were collected from the mothers’ and newborns’ medical charts. Four health professionals (three BSc midwifery data collectors) and one MSc supervisor were recruited. The qualities of the data were ensured by designing the checklist properly from different studies. Pretest of the checklist was conducted on 5% of the sample at ACSH before going to the actual data collection. Two days of training were given to the data collectors and supervisors by the principal investigator regarding the objective of the study, the data collection process, the purpose of the study, and ethical issues and the data were cleaned before analysis. The reliability of the data was checked in SPSS via Cronbach’s Apha and a value greater than 0.6 was considered acceptable. The reliability test result was 0.61, which is reliable.

### Data processing and analysis

The collected data were entered, coded, cleaned with SPSS version 25 and then exported to STATA version 14 software for analysis. Descriptive statistics such as frequencies and percentages for categorical variables and summary statistics for continuous data (median with IQR) were used to describe the study populations. The normality distribution of continuous variables was tested using the Shapiro-Wilk tests. A *p*-value < 0.05 indicating that the data were not normally distributed. Linearity was checked using a lack fit test, and a *p*-value of 0.023 indicated that linear regression was not appropriate.

To estimate relative risks for common binary outcomes, log binomial regression and modified Poisson regression are commonly used [21]. Since log-binomial regression often faces convergent problem modified Poisson regression was used in this study. Modified Poisson regression, which combines a log Poisson regression model with robust standard error, is useful for estimating relative risks [22]. A modified Poisson regression model was used to estimate relative risks to identify the effect of antepartum hemorrhage on adverse maternal and perinatal outcomes. Independent variables with a *p*-value < 0.25 at the bivariate regression were included in the multivariable modified Poisson regression analysis (final model). Maternal age, residence, history of stillbirth, history of cesarean section, gravidity, parity, ANC visit, and mode of delivery were controlled for in the final model. After adjusting for other variables, the relative risk with 95% CI and a *p*-value < 0.05 was considered statistically significant. Multicollinearity among independent variables was checked using variance inflation factor (VIF), with a mean (VIF) of less than 5, indicating no multicollinearity existed in the data. The results are in texts and tables.

## Results

### Sociodemographic and obstetric characteristics of the study participants

**Fig. 1:**
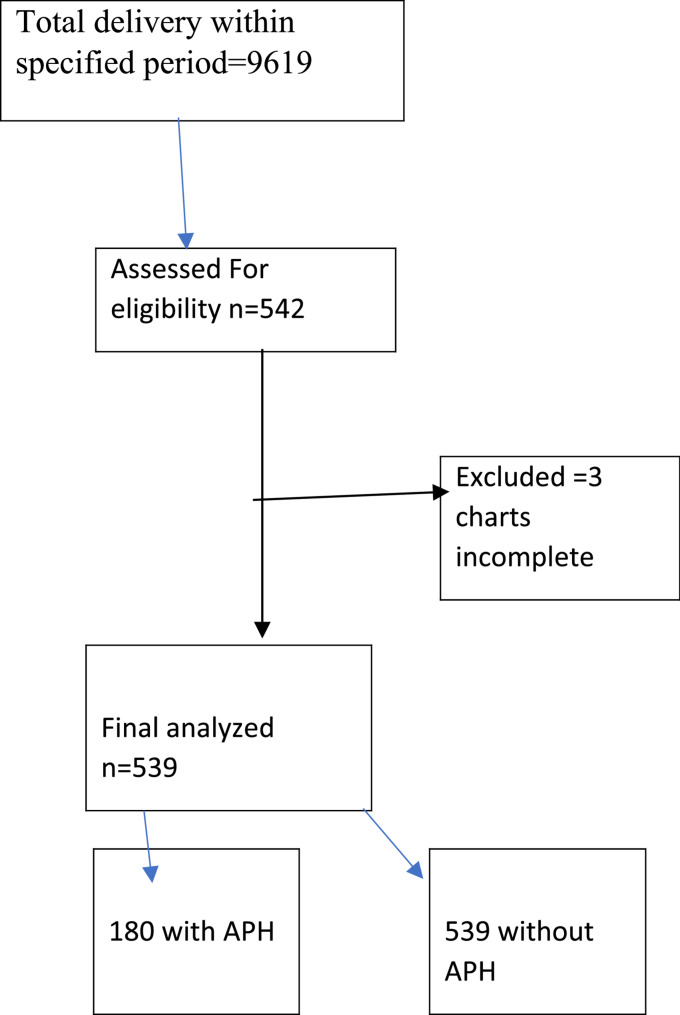
Schematic presentation of sampling procedure of study participant at ACSH, Mekelle, Tigray, Ethiopia 2024

A total of 539 medical charts of mothers who delivered at ACSH were reviewed. Among these, 180 were mothers with antepartum hemorrhage and 359 mothers were without antepartum hemorrhage (Fig. 1). Among mothers with APH, 50.0% had abruption placenta, 48.9% had placenta previa and 1.1% had extraplacental causes. The median age (interquartile range) was 28 (25–35) years for mothers with APH and 26 (23–31) years for mothers without APH. More than half of the mothers’ records (52.2% of mothers with APH and 55.7% without APH) were in the age range of 25–34 years. With respect to obstetric characteristics:- 55.0% of mothers with APH and approximately 50.8% of mothers without APH had two to four histories of pregnancy including the current one. In terms of antenatal care (ANC) visits, 92.2% of mothers with APH and 97.2% of mothers without APH had at least one ANC visit (Table 1).

**Table 1:** Sociodemographic characteristics of the study participants Tigray, Ethiopia 2024

Variable	Response	With APH *n* (%)	Without APH *n* (%)	Total *n* (%)
Age	15–24	39 (21.7)	113 (31.5)	152 (28.2)
	25–34	94 (52.2)	200 (55.7)	294 (54.5)
	$\:\ge\:$35	47 (26.1)	46 (12.8)	93 (17.3)
Residence	Urban	135 (75.0)	294(81.9)	429 (79.6)
	Rural	45 (25.0)	65 (18.1)	110 (20.4)
Gravidity	1	37 (20.6)	141 (39.3)	178 (33.0)
	2–4	99 (55.0)	175 (48.8)	274 (50.8)
	$\:\ge\:$5	44 (24.4)	43 (19.9)	87 (16.1)
Parity	Primipara	43 (23.9)	165 (45.9)	208 (38.6)
	Multipara	137 (76.1)	194 (54.0)	331 (61.4)
ANC visit	Yes	166(92.2)	349 (97.2)	515 (95.6)
	No	14 (7.8)	10(2.8)	24(4.4)

### Incidence of adverse maternal outcomes among study participants

The incidence of adverse maternal outcomes among mothers with antepartum hemorrhage was 83(46.1%), while among mothers without APH, it was 51(14.2%). There were significant differences in all adverse maternal outcomes between the two groups. Of the total mothers with APH, 78(43.3%) delivered by emergency cesarean section, whereas approximately 49(13.7%) mothers without APH delivered by emergency cesarean section. Additionally, 19(10.6%) mothers with APH developed postpartum hemorrhage (PPH), compared to 8(2.2%) mothers without APH (Table 2).

**Table 2:** Incidence of adverse maternal outcomes among study participants tigray, Ethiopia 2024

Variables	Response	With APH	without APH	Total	*P* value
		n (%)	n (%)	n (%)	
PPH	Yes	19(10.6)	8(2.2)	27(5.1)	
	No	161 (89.4)	351 (97.7)	512 (94.9)	< 0.001*
Emergency c/s	Yes	78(43.3)	49(13.7)	127 (23.6)	< 0.001*
	No	102 (56.7)	310 (86.3)	412(76.4)	
Blood transfusion	Yes	15(8.3)	3()	18(3.3)	< 0.001**
	No	165 (91.7)	356 (99.2)	521 (96.7)	
ICU admission	Yes	5(2.8)	2	7 (1.3)	0.032**
	No	175(97.2)	357(99.4)	532 (98.7)	
adverse maternal outcome	Yes	83 (46.1)	51(14.2)	134 (24.9)	
	No	97(53.9)	308 (85.8)	405 (75.1)	< 0.001*

*Pearson chi square test

**Fisher’s exact test

### Incidence of adverse perinatal outcomes among study participants

More than half of the 103(57.2%) mothers with APH, and 68(18.9%) of mothers without APH developed adverse perinatal outcomes. Mothers with APH experienced 83(46.1%) preterm births, whereas mothers without APH experienced 41(11.4%) preterm births. Regarding the birth weight of the newborn, there was high incidence of low birth weight among mothers with APH, 75(41.7%), compared to 32(8.91%) mothers without APH. In addition, 40(22.2%), 27(15%) of babies born from mothers with APH had low Apgar scores at the first and 5th minutes, compared to, 27(7.5%), 14(3.9%) from mothers without APH, respectively. The incidence of perinatal death among mothers with APH and without APH was (17.2% and 4.5% respectively), (Table 3).

**Table 3:** Incidence of adverse perinatal outcomes among study participants, tigray, Ethiopia 2024

Variables	Response	With APH	without APH	Total	*P* value
		n (%)	n (%)	n (%)	
Preterm birth	Yes	83 (46.1)	41 (11.4)	124(23.0)	< 0.001*
	No	97 (53.9)	318 (88.6)	415 (77.0)	
LBW	Yes	75 (41.7)	32 (8.9)	107 (19.9)	< 0.001*
	No	105 (58.3)	327 (91.1)	432 (80.1)	
Still birth	Yes	25 (13.9)	13 (3.6)	38 (7.0)	< 0.001*
	No	155 (86.1)	346 (96.4)	501 (93.0)	
Perinatal death	Yes	31 (17.2)	16 (4.5)	47 (8.7)	< 0.001*
	No	149 (82.8)	343 (95.5)	482 (91.3)	
NICU admission	Yes	28 (15.6)	9 (2.5)	37 (6.9)	< 0.001*
	No	152 (84.4)	350 (97.5)	4 (93.1)	
Low Apgar score 1 st minute	Yes	40 (22.2)	27 (7.5)	67 (12.4)	< 0.001*
	No	140 (77.8)	332 (92.5)	472 (87.6)	
Low Apgar score at 5th Minute	Yes	27 (15.0)	14(3.9)	41 (7.6)	< 0.001*
	No	153 (85.0)	345 (96.1)	498 (92.4)	
Adverse perinatal outcome	Yes	103 (57.2)	68(18.9)	171 (31.7)	< 0.001*
	No	77 (42.8)	291 (81.1)	368 (68.3)	

*Pearson chi square test

### Risk of each adverse maternal outcome associated with APH

APH was significantly associated with higher adverse maternal outcomes, mothers with APH were more likely to experience postpartum hemorrhage (ARR = 3.9, 95% CI: 1.8, 8.8), and emergency cesarean section (ARR = 2.9, 95% CI: 2.1, 3.9) compared to mothers without APH (Table 4).

**Table 4:** Bivariate and multivariate modified Poisson regression analyses of the associations of APH with adverse maternal outcomes tigray, ethiopia, 2024

Variables	Response	Postpartum hemorrhage		CRR (95%CI)	ARR (95% CI)
		Yes (*n* = 27) n (%)	No(*n* = 512) n (%)		
Age	15–24	5(18.5)	149(29.1)	1	1
	25–34	16(59.3)	276(53.9)	3.1(0.9, 10.4)	2.41(0.7, 8.4)
	$\:\ge\:$35	6(22.2)	87(17.0)	3.3(0.8, 12.8)	1.8(0.4, 7.8)
Parity	Primipara	5(18.5)	203(39.6)	1	1
	Multipara	22(81.5)	309(60.4)	2.8(1.1, 7.2)*	1.6(0.6, 4.5)
ANC	Yes	23(85.2)	492(96.1)	1	1
	No	4(14.8)	20(3.9)	3.7(1.4, 9.9)	2.7(1.0, 7.3)
APH	Yes	19(70.4)	161(31.4)	4.7(2.1, 10.6)*	3.9(1.8, 8.8)*
	No	8(29.4)	351(68.6)	1	1
		Emergency cesarean section			
		Yes (*n* = 127) n (%)	No(*n* = 412) n (%)		
Residence	Urban	95(74.8)	334(81.1)	1	1
	Rural	32(25.2)	78(18.9)	1.3(0.9, 1.9)	0.9(0.7, 1.3)
Age	15–24	25(19.7)	127(30.8)	1	1
	25–34	76(59.8)	218(52.9)	1.6(1.1, 2.4)*	1.4(0.9, 2.0)
	$\:\ge\:$35	26(20.5)	67(16.3)	1.7(1.1, 2.8)*	1.15(0.7, 1.9)
Parity	Primipara	34(26.8)	174(42.2)	1	1
	Multipara	93(73.2)	238(57.8)	1.7(1.2, 2.5)*	1.2(0.9, 1.8)
ANC	Yes	111(87.4)	404(98.1)	1	1
	No	16(12.6)	8(1.9)	3.1(2.2, 4.3)*	2.4(1.8, 3.4)*
APH	Yes	78(61.4)	102(24.8)	3.2(2.3, 4.3)*	2.9(2.1, 3.9)*
	No	49(38.6)	310(75.2)	1	1

*CRR *crude relative risk, *ARR *adjusted relative risk, *CI *confidence interval

*p<0.05

### Risk of each adverse perinatal outcome associated with APH

In the final multivariable modified Poisson regression analysis APH was significantly associated with higher adverse perinatal outcomes, mothers with APH were more likely to experience preterm birth (ARR = 3.9, 95%CI: 2.8, 5.6), low birth weight baby (ARR = 4.5, 95%CI: 3.0, 6.6), stillbirth (ARR = 3.8, 95%CI: 1.9, 7.4), perinatal death (ARR = 3.7, 95%CI: 2.0, 6.9), admission to the NICU (ARR = 6.7, 95% CI: 3.1, 14.9), low Apgar score at the first minute (ARR = 2.8, 95%CI: 1.8, 4.3), and low Apgar score at the fifth minute (ARR = 3.7, 95% CI: 2.0, 6.8) compared to mothers without APH (Table 5).

**Table 5:** Bivariate and multivariate modified Poisson regression analyses of the associations of APH with each adverse perinatal outcome, tigray, ethiopia, 2024

Variables	Response	Preterm birth		CRR (95% CI)	ARR (95% CI)
		Yes (124) *n* (%)	No (415) *n* (%)		
Residence	Urban	91(73.4)	338(81.4)	1	1
	Rural	33(26.6)	77(18.6)	1.4(1.0, 1.1)*	1.1(0.8, 1.5)
Gravidity	1	36(29.1)	142(34.2)	0.9(0.6, 1.3)	1.2(0.8, 1.6)
	2–4	61(49.2)	213(51.3)	1	1
	$\:\ge\:$5	27(21.8)	60(14.5)	1.4(0.9, 2.1)*	1.1(0.9, 1.6)
ANC	Yes	111(89.5)	404(97.3)	1	1
	No	13(10.5)	11(2.7)	2.5(1.7, 3.8)	**1.7**(1.0, 2.7)*
APH	Yes	83(66.9)	97(23.4)	4.0(2.9, 5.6)	**3.9**(2.8, 5.6)*
	No	41(33.1)	318(76.6)	1	1
**Low birth weight**					
		Yes (107) n(%)	No (432) n(%)		
Age	15–24	28(26.2)	124(28.7)	1	1
	25–34	56(52.3)	238(55.1)	1.0(0.7, 1.6)	0.9 (0.6, 1.3)
	$\:\ge\:$35	23(21.5)	70(16.2)	1.3(0.8, 2.2)	0.9(0.6, 1.5)
Residence	Urban	78(72.9)	351(81.2)	1	1
	Rural	29(27.1)	81(18.8)	1.5(1.0, 2.1)	1.1(0.8, 1.6)
ANC	Yes	96(89.7)	419(97.0)	1	1
	No	11(10.3)	13(3.0)	2.5(1.5, 3.9)	1.6(0.9, 2.7)
APH	Yes	75(70.1)	105(24.3)	4.7(3.2, 6.8)	**4.5**(3.0, 6.6)*
	No	32(29.9)	327(75.7)	1	1
**Still birth**					
		Yes (38) n(%)	No (501) n(%)		
Age	15–24	9(23.7)	143(28.5)	1	1
	25–34	18(47.4)	276(55.1)	1.0(0.5, 2.3)	1.0(0.5, 2.4)
	$\:\ge\:$35	11(28.9)	82(16.4)	1.9(0.7, 4.6)*	1.8(0.6, 5.2)
Gravidity	1	11(28.9)	167(33.3)	0.9(0.5, 2.0.)	1.3(0.6, 2.9)
	2–4	18(47.4)	256(51.1)	1	1
	$\:\ge\:$5	9(23.7)	78(15.6)	1.6(0.7, 3.4)	0.9(0.4, 2.3)
APH	Yes	25(65.8)	155(30.9)	3.8(2.0, 7.3)	**3.8**(1.9, 7.4)*
	No	13(34.2)	346(69.1)	1	1
**Perinatal death**					
		Yes (47) n(%)	No (492) n(%)		
Age	15–24	12(25.5)	140(28.5)	1	1
	25–34	23(49.0)	271(55.1)	0.9(0.5, 1.9)	0.9(0.5, 1.9)
	$\:\ge\:$35	12(25.5)	81(16.4)	1.6(0.8, 3.5)*	1.2(0.5, 3.3)
Residence	Urban	32(68.1)	397(80.7)	1	1
	Rural	15(31.9)	95(19.3)	1.8(1.0, 3.3)	1.6(0.9, 2.9)
Gravidity	1	13(27.7)	165(33.5)	0.9(0.5, 1.7)	1.2(0.6, 2.4)
	2–4	23(48.9)	251(51.0)	1	1
	$\:\ge\:$5	11(23.4)	76(15.4)	1.5(0.8, 3.0)	0.9(0.4, 2.4)
APH	Yes	31(66.0)	149(30.3)	3.9(2.2, 6.9)	**3.7**(2.0, 7.0)*
	No	16(34.0)	343(69.7)	1	1
**1** ^st^ **minute low Apgar score**					
		Yes (67) n(%)	No (472)n(%)		
Residence	Urban	41(61.2)	388(82.2)	1	1
	Rural	26(38.8)	84(17.8)	2.5(1.6, 3.7)**	**2.2**(1.5, 3.4) *
APH	Yes	40(59.7)	140(29.7)	2.9(1.9, 4.7) **	**2.76**(1.8, 4.3)*
	No	27(40.3)	332(30.3)	1	1
**5** ^th^ **minute low Apgar score**					
		Yes (41)n(%)	No (498) n(%)		
Residence	Urban	27(65.9)	402(80.7)	1	1
	Rural	14(34.1)	96(19.3)	2.0(1.1, 3.7)	1.9(0.9, 3.2)
APH	Yes	27(65.9)	153(30.7)	3.9(2.0, 7.2)	**3.7**(1.9, 6.8)*
	No	14(34.1)	345(69.3)	1	1
**Admission to NICU**					
		Yes (37) n(%)	No (502) n(%)		
Age	15–24	12(32.4)	140(27.9)	1	1
	25–34	22(59.5)	272(54.2)	0.9(0.5, 1.9)	0.8(0.4, 1.6)
	$\:\ge\:$35	3(8.1)	90(17.9)	0.4(0.1, 1.4)	**0.3**(0.1, 0.9)*
ANC	Yes	33(89.2)	482(96.0)	1	1
	No	4(10.8)	20(4.0)	2.6(1.0, 6.8)	1.6(0.7, 4.7)
APH	Yes	28(75.7)	152(30.3)	6.2(3.0, 12.9)	**6.7**(3.1, 14.9)*
	No	9(24.3)	350(69.7)	1	1

*CRR* crude relative risk, *ARR* adjusted relative risk, *CI* confidence interval, Bold=significant variables *p*-value <0.05

*p<0.05

## Discussion

This study aimed to assess the effects of antepartum hemorrhage on adverse maternal and perinatal outcomes. On the basis of these finding, mothers with APH had a higher risk of adverse maternal and perinatal outcomes, such as postpartum hemorrhage, emergency cesarean section, low birth weight, preterm birth, stillbirth, admission to the NICU, low Apgar score, and perinatal death.

The findings of this study revealed that mothers with antepartum hemorrhage were 3.9 times more likely to be at risk of experiencing postpartum hemorrhage than mothers without antepartum hemorrhage. This result aligns with studies conducted in South Korea [15], Northern Tanzania [17, 19], which also revealed a significant association between APH and an increased risk of PPH. Similarly, a study conducted in Ethiopia reported that mothers with APH were 4.8 times more likely to develop sever PPH compared to those without APH [23]. This similarity might be due to placental problems such as placenta previa and placenta abruption, which are major the risk factor for postpartum hemorrhage and affect the ability of the uterus to contract after delivery, resulting in bleeding [24]. However, these findings contradict a study done in Ethiopia at Tikur Anbesa Specialized and Gandhi Memorial Hospital [14]. This difference might be due to the differences in the causes of APH considered. This study includes the causes of APH such as placenta previa and abruptio placenta and done only in one referral hospital, but the study in Ethiopia; Tikur Anbesa Specialized and Gandhi Memorial Hospital was focused on placenta previa and conducted in two hospitals.

Another finding of this study was that mothers with antepartum hemorrhage were 2.9 times more likely to undergo emergency cesarean section than mothers without APH. This result is consistent with the studies conducted in China [25] and South Korea [15]. Likewise, a study from Addis Ababa university reported that 62.5% of women with APH underwent cesarean Sect. [26]. This association could be explained by the urgent clinical need to minimize risks to both the mother and fetus when bleeding occurs during pregnancy [26]. Women with APH and associated maternal and/or fetal compromise are required to be delivered immediately, and a cesarean section is the appropriate method of delivery with resuscitation of the mother [27].

The findings of this study showed that mothers with antepartum hemorrhage were 4.5 times more likely to have low birth weight babies. This finding is similar to that of a studies conducted in Northern Tanzania [17, 19]. Additionally, a multi country study across Africa, Asia, and Latin America identified sever APH as a major risk factor for low birth weight [28]. The increased risk of low birth weight among mothers with APH could be, APH affects the uteroplacental blood flow, leading to intrauterine growth restriction, the main cause of low birth weight [29]. However, this finding contrasts with study done in Ethiopia, Tikur Anbesa Specialized and Gandhi Memorial Hospital [14]. The difference across studies could be due to the differences in the quality of antenatal care services and the management of antepartum hemorrhage across the study areas.

This study also revealed that mothers with antepartum hemorrhage were 3.9 times more likely to have preterm birth babies. This finding aligns with studies conducted in Kenya and Ethiopia [14, 20]. Furthermore, a systematic review and meta-analysis conducted in Ethiopia reported that women with APH were five times more likely to give preterm birth [30]. Similarly, a study conducted at Felege Hiwot Comprehensive Specialized Referal Hospital, Northwest Ethiopia found that mothers with a history of APH had 3.53 times higher odds of experiencing preterm birth [31]. The increased risk of delivering preterm birth among mother with APH than mother without APH, this may be due to the life-threatening nature of APH, necessitating pregnancy termination, despite gestational age, for maternal or fetal indications [32] and spontaneous labor triggered by reduced blood flow and nutrient supply to the fetus can initiate preterm labor [29]. However, this finding is inconsistent with study in Northern Tanzania [17]. The justification for the disparity might be due to differences in the study setting and the quality of antenatal care services.

Furthermore, this study revealed that babies born from mothers with APH were 3.7 times more likely to have a low 5th minute Apgar score compared to babies born from mothers without APH. This result is consistent with the studies conducted in Kenya [20], Northern Tanzania [17, 19], in North Shoa, Oromia, Ethiopia [33], and a systematic and meta-analysis in Ethiopia [34]. The review suggested that APH contributes to low Apgar scores indirectly by increasing the risk of preterm birth and low birth both of which are common consequences of APH and major contributors to lower Apgar scores at birth [34]. The high risk of a low Apgar score might be due to prematurity, with preterm infants being vulnerable to immaturity of muscle tone and reflex irritability. The lungs of preterm infants may be deficient in surfactant, which makes the lungs more difficult to ventilate [35]. For this reason, all necessary equipment for newborn resuscitation should be ready at every delivery by anticipating the risk of birth with a low Apgar score among women with APH.

Additionally, this study showed that APH was significantly associated with stillbirth, perinatal death, and admission to the NICU. This finding is consistent with finding from Northern Tanzania [17, 19], Kenya [20]. Similarly, a study conducted at Hiwot Fana Specialized University Hospital, in Harar, Eastern Ethiopia, reported that mothers with history of APH were 3.3 times more likely to experience still birth compared to without history of APH [36]. The increased risk of stillbirth among mothers with APH might be related to APH leads to decreased uteroplacental blood flow and placental ischemia, which compromises blood flow to the fetus [29]. High risk of perinatal death caused by prematurity and intrauterine fetal growth restriction. The high risk of newborn babies being admitted to the NICU among newborns delivered from women with APH compared with those from women without APH might be related to increased incidence of preterm birth, and increased numbers of babies with low birth weights as an adverse effect of antepartum hemorrhage [37].

These findings suggested that APH is significantly associated with adverse maternal and perinatal outcomes. Therefore, policymakers and programmers should use this evidence to identify pregnant women at high risk for APH during antenatal care visits, which may help with the early identification and management of APH and access to emergency obstetric care. The Tigray Regional Health Bureau and district health offices should also use this evidence to improve maternal and perinatal health outcomes in collaboration with other stakeholders. In addition, health care providers should strengthen early diagnosis and management of antepartum hemorrhage, provide services to manage APH and facilitate a referral system for the immediate transfer of patients to higher-level facilities in case of emergencies to reduce the incidence of adverse maternal and perinatal outcomes of antepartum hemorrhage. Other researchers need to include variables measured via prospective methods.

The strength of this study is that it is a comparative study that addresses the associations of antepartum hemorrhage with adverse maternal and perinatal outcomes. As a limitation since this study was used secondary data, variables that might have possible relationships with adverse maternal and perinatal outcomes, such as maternal nutritional status, smoking history, maternal educational status, and income level were not available. Therefore, hospital record keeping should be improved. Additionally, since this was hospital-based study, the results may not be generalized e to the whole population. Adverse outcomes occurring after hospital discharge and home deliveries were not addressed.

## Conclusions

Antepartum hemorrhage is associated with a higher risk of adverse maternal and perinatal outcomes such as postpartum hemorrhage, emergency cesarean section, a higher risk of preterm birth, low birth weight, stillbirth, perinatal death, a low Apgar score, and admission to the NICU. Early identification of high-risk pregnancies through regular screening is essential to identify at risk women and ensure they receive timely care. Furthermore, access to emergency obstetric care in areas with a high burden of APH is critical to ensure timely intervention, which can reduce adverse maternal and perinatal outcomes.

## Supplementary Information

Supplementary Material 1.

## Data Availability

The datasets used and analyzed during this study are available from the corresponding author upon reasonable request.

## Abbreviations

ACSH: Ayder Comprehensive Specialized Hospital
APGAR: Appearance Pulse Grimace Activity Respiration
APH: Antepartum Hemorrhage
ANC: Antenatal Care
ICU: Intensive Care Unit
LBW: Low birth Weight
NICU: Neonatal Intensive Care Unit
PPH: Postpartum Hemorrhage
SPSS: Statistics Package for Social Science
VIF: Variance Inflation Factor
