# Supplementary material for: Effects of antepartum hemorrhage on maternal and perinatal adverse outcomes in Northern Ethiopia: a retrospective cohort study
Source: BMC Pregnancy Childbirth. 2025 Jul 22;25:781. doi: 10.1186/s12884-025-07829-0 (PMC12281754; doi:10.1186/s12884-025-07829-0)
Supplement: Supplementary file 1 — Supplementary Material 1. [file 12884_2025_7829_MOESM1_ESM.docx]

**Additional file 1:** Bivariate and multivariable modified poisson regression analysis on association of APH with each adverse perinatal outcomes, Tigray, Ethiopia, 2024.

| Variables | Response | Preterm birth | | | CRR (95% CI) | ARR (95% CI) |
| --- | --- | --- | --- | --- | --- | --- |
|  |  | Yes (124) | No (415) | |  |  |
|  |  | n (%) | n (%) | |  |  |
| Residence | Urban | 91(73.4) | 338(81.4) | | 1 | 1 |
|  | Rural | 33(26.6) | 77(18.6) | | 1.4(1.0, 1.1)** | 1.1(0.8, 1.5) |
| Gravidity | 1 | 36(29.1) | 142(34.2) | | 0.9(0.6, 1.3) | 1.2(0.83, 1.6) |
|  | 2 – 4 | 61(49.2) | 213(51.3) | | 1 | 1 |
|  | $\boldsymbol{\geq}$ 5 | 27(21.8) | 60(14.5) | | 1.4(0.9, 2.1)* | 1.1(0.88, 1.6) |
| ANC | Yes | 111(89.5) | 404(97.3) | | 1 | 1 |
|  | No | 13(10.5) | 11(2.7) | | 2.5(1.7, 3.8) | **1.7**(1.0, 2.7)* |
| APH | Yes | 83(66.9) | 97(23.4) | | 4.0(2.9, 5.6) | **3.9**(2.8, 5.6)* |
|  | No | 41(33.1) | 318(76.6) | | 1 | 1 |
|  |  | **Low birth weight** | | |  |  |
|  |  | Yes (107)  n(%) | | No (432)  n(%) |  |  |
| Age | 15 -24 | 28(26.2) | | 124(28.7) | 1 | 1 |
|  | 25- 34 | 56(52.3) | | 238(55.1) | 1.0(0.7, 1.6) | 0.9 (0.6, 1.3) |
|  | $\boldsymbol{\geq}$ 35 | 23(21.5) | | 70(16.2) | 1.3(0.8, 2.2) | 0.9(0.6, 1.5) |
| Residence | Urban | 78(72.9) | | 351(81.2) | 1 | 1 |
|  | Rural | 29(27.1) | | 81(18.8) | 1.5(1.0, 2.1) | 1.1(0.8, 1.6) |
| ANC | Yes | 96(89.7) | | 419(97.0) | 1 | 1 |
|  | No | 11(10.3) | | 13(3.0) | 2.5(1.5, 3.9) | 1.6(0.9, 2.7) |
| APH | Yes | 75(70.1) | | 105(24.3) | 4.7(3.2, 6.8) | **4.5**(3.0, 6.6)* |
|  | No | 32(29.9) | | 327(75.7) | 1 | 1 |
|  |  | **Still birth** | | |  |  |
|  |  | **Yes (38)**  **n(%)** | | **No (501)**  **n(%)** |  |  |
| Age | 15 - 24 | 9(23.7) | | 143(28.5) | 1 | 1 |
|  | 25 - 34 | 18(47.4) | | 276(55.1) | 1.0(0.5, 2.3) | 1.0(0.5, 2.4) |
|  | $\boldsymbol{\geq}$ 35 | 11(28.9) | | 82(16.4) | 1.9(0.7, 4.6)* | 1.8(0.6, 5.2) |
| Gravidity | 1 | 11(28.9) | | 167(33.3) | 0.9(0.5, 2.0.) | 1.3(0.6, 2.9) |
|  | 2 - 4 | 18(47.4) | | 256(51.1) | 1 | 1 |
|  | $\boldsymbol{\geq}$ 5 | 9(23.7) | | 78(15.6) | 1.6(0.7, 3.4) | 0.9(0.4, 2.3) |
| APH | yes | 25(65.8) | | 155(30.9) | 3.8(2.0, 7.3) | **3.8**(1.9, 7.4)* |
|  | No | 13(34.2) | | 346(69.1) | 1 | 1 |
|  |  | **Perinatal death** | | |  |  |
|  |  | Yes (47)  n(%) | | No (492)  n(%) |  |  |
| Age | 15 - 24 | 12(25.5) | | 140(28.5) | 1 | 1 |
|  | 25 - 34 | 23(49.0) | | 271(55.1) | 0.9(0.5, 1.9) | 0.9(0.5, 1.9) |
|  | $\boldsymbol{\geq}$ 35 | 12(25.5) | | 81(16.4) | 1.6(0.8, 3.5)* | 1.2(0.5, 3.3) |
| Residence | Urban | 32(68.1) | | 397(80.7) | 1 | 1 |
|  | Rural | 15(31.9) | | 95(19.3) | 1.8(1.0, 3.3) | 1.6(0.9, 2.9) |
| Gravidity | 1 | 13(27.7) | | 165(33.5) | 0.9(0.5, 1.7) | 1.2(0.56, 2.36) |
|  | 2 - 4 | 23(48.9) | | 251(51.0) | 1 | 1 |
|  | $\boldsymbol{\geq}$ 5 | 11(23.4) | | 76(15.4) | 1.5(0.8, 3.0) | 0.9(0.4, 2.4) |
| APH | Yes | 31(66.0) | | 149(30.3) | 3.9(2.2, 6.9) | **3.7**(2.0, 7.0)* |
|  | No | 16(34.0) | | 343(69.7) | 1 | 1 |
|  |  | **1^st^minute low Apgar score** | | |  |  |
|  |  | Yes (67)  n(%) | | No (472)  n(%) |  |  |
| Residence | Urban | 41(61.2) | | 388(82.2) | 1 | 1 |
|  | Rural | 26(38.8) | | 84(17.8) | 2.5(1.6, 3.7)** | **2.2**(1.5, 3.4)* |
| APH | Yes | 40(59.7) | | 140(29.7) | 2.9(1.9, 4.7)** | **2.76**(1.8, 4.3)* |
|  | No | 27(40.3) | | 332(30.3) | 1 | 1 |
|  |  | **5^th^minute low Apgar score** | | |  |  |
|  |  | Yes (41)  n(%) | No (498)  n(%) | |  |  |
| Residence | Urban | 27(65.9) | 402(80.7) | | 1 | 1 |
|  | Rural | 14(34.1) | 96(19.3) | | 2.0(1.1, 3.7) | 1.9(0.9, 3.2) |
| APH | Yes | 27(65.9) | 153(30.7) | | 3.9(2.0, 7.2) | **3.7**(1.9, 6.8)* |
|  | No | 14(34.1) | 345(69.3) | | 1 | 1 |
|  |  | **Admission to NICU** | | |  |  |
|  |  | Yes (37)  n(%) | No (502)  n(%) | |  |  |
| Age | 15 -24 | 12(32.4) | 140(27.9) | | 1 | 1 |
|  | 25- 34 | 22(59.5) | 272(54.2) | | 0.9(0.5, 1.9) | 0.8(0.4, 1.6) |
|  | $\boldsymbol{\geq}$ 35 | 3(8.1) | 90(17.9) | | 0.4(0.1, 1.4) | **0.3(**0.1, 0.9)* |
| ANC | Yes | 33(89.2) | 482(96.0) | | 1 | 1 |
|  | No | 4(10.8) | 20(4.0) | | 2.6(1.0, 6.8) | 1.6(065, 4.7) |
| APH | Yes | 28(75.7) | 152(30.3) | | 6.2(3.0, 12.9) | **6.7**(3.1, 14.9)* |
|  | No | 9(24.3) | 350(69.7) | | 1 | 1 |

* p<0.05. CRR= crude relative risk, ARR = adjusted relative risk, CI=confidence interval.

**Additional file 2. Association of placenta previa and abruption placenta with adverse maternal outcomes, Tigray, Ethiopia,2024.**

| Variables | Response | Placenta previa | | | p-value |
| --- | --- | --- | --- | --- | --- |
|  |  | Yes | No | Total |  |
| PPH | Yes | 15 | 4 | 19 | 0.007* |
|  | No | 73 | 88 | 161 |  |
| Emergency C/S | Yes | 51 | 27 | 78 | <0.001* |
|  | No | 37 | 65 | 102 |  |

| Variables | Response | Abruptio placenta | | | p-value |
| --- | --- | --- | --- | --- | --- |
|  |  | Yes | No | Total |  |
| PPH | Yes | 4 | 15 | 19 | 0.007* |
|  | No | 86 | 75 | 161 |  |
| Emergency C/S | Yes | 26 | 52 | 78 | <0.001* |
|  | No | 64 | 38 | 102 |  |

***: Pearson chi square test.**
